# Acantholytic Pityriasis Rubra Pilaris Associated with Imiquimod 3.75% Application

**DOI:** 10.1155/2011/412684

**Published:** 2011-10-31

**Authors:** Natasha Atanaskova Mesinkovska, Danyelle Dawes, Apra Sood, Wilma Bergfeld

**Affiliations:** ^1^Department of Dermatology, Cleveland Clinic, Cleveland, OH 44195, USA; ^2^Case Western Reserve University School of Medicine, Cleveland, OH 44106, USA

## Abstract

Imiquimod is an immunomodulator with both antitumor and antiviral properties. It is currently available in two cream formulations as Aldara (imiquimod 5%) and the newly approved Zyclara (imiquimod 3.75%). Imiquimod has been associated with localized erythema, crusting, and scaling at the site of application. However, more severe generalized skin eruptions including erythema multiforme, psoriasis, and hyperpigmentation have been described. The newly approved imiquimod 3.75% cream is a presumably safer alternative due to its lower concentration. This paper describes the development of generalized acantholytic pityriasis rubra pilaris after the treatment of an actinic keratosis on the forehead with imiquimod 3.75% cream.

## 1. Report

A 65-year-old Caucasian male with a past medical history of hypertension and hyperlipidemia was diagnosed with actinic keratosis on his left temple by his dermatologist. He was instructed to apply imiquimod 3.75% cream on the involved area once daily for 6 weeks (2 weeks on, 2 weeks off, 2 weeks on). During the fifth week of treatment, he developed a painful, pruritic, erythematous plaque at the site of application and discontinued the imiquimod 3.75% cream. Over the next several days, the plaque on his left temple expanded to involve his entire face. He also developed pruritic, erythematous plaques on his neck, trunk, and extremities. He denied any systemic symptoms such as fever, malaise, or lymphadenopathy. Treatment with oral prednisone and antihistamines did not stop the progression of his dermatitis nor improve his symptoms. 

**Figure 1:**
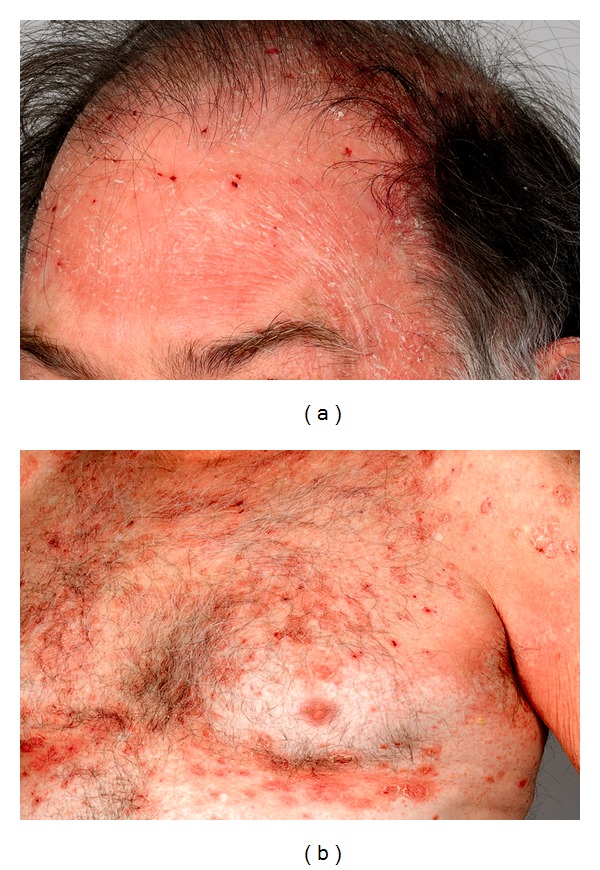
(a) Salmon-colored erythematous plaques with superficial thin scales involving the left temple site where imiquimod 3.75% was applied and extending over forehead, scalp, and ear. (b) Small, perifollicular, salmon-colored papules coalesce to form a background of erythema, with islands of sparing over chest. There are overlaying, prominent, erythematous oval plaques with flaky scale.

The patient presented to our clinic one month after the development of his skin lesions. On examination, he had salmon-colored erythematous plaques with superficial thin scales covering his face, scalp, and neck (Figure 1(a)). Similar lesions were noted on his trunk and extremities, some coalescing to form large areas of erythema (Figure 1(b)). There were sharply demarcated islands of normal unaffected skin on his chest. Both palms were hyperkeratotic and erythematous with some mild ridging of the nails. Examinations of oral and genital mucosa were unremarkable. There was no personal or family history suggestive of skin or connective tissue disease. At first glance, the clinical picture was suggestive of pemphigus foliaceus, but the “islands” of unaffected skin and his hyperkeratotic palms were consistent with pityriasis rubra pilaris (PRP). Blood counts and complete metabolic panel were within normal limits, and antistreptolysin (ASO) antibodies were negative. A biopsy sample was taken from one of the scaly erythematous plaques on the left side of the abdomen.

**Figure 2:**
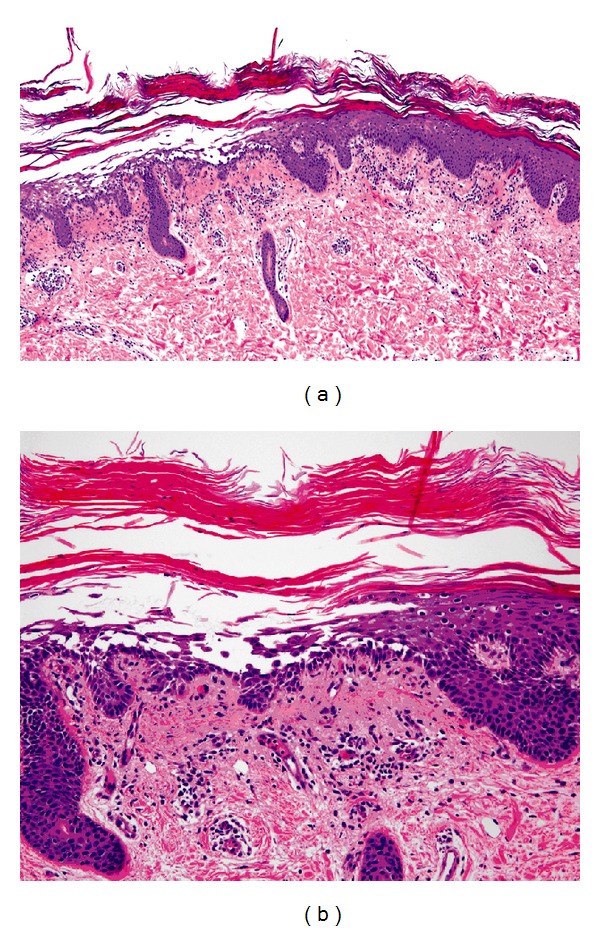
Skin biopsy, left abdomen. Alternating parakeratosis and orthokeratosis in the stratum corneum in vertical and horizontal sections. A focus of acantholysis in the epidermis with suprabasilar and subcorneal location. Within the superficial dermis, there is a sparse superficial perivascular and interstitial lymphocytic infiltrate. Hematoxylin-eosin stain (a) ×4 and (b) ×20 magnification.

Histopathologic sections revealed alternating parakeratosis and orthokeratosis in both the vertical and horizontal directions and a mildly acanthotic epidermis with confluent hypergranulosis (Figure 2(a)). The epidermis had multiple small foci of acantholysis, located at suprabasilar or subcorneal levels (Figure 2(b)). There was a very sparse superficial perivascular and interstitial lymphocytic infiltrate in the dermis. Direct immunofluorescence studies of the acantholytic areas were negative. Upon review of identical findings on a repeat biopsy, it was agreed that the diagnosis was consistent with the rare acantholytic variant of PRP.

Therapeutic management of this patient was challenging and was further complicated by his reluctance to use systemic medications. He was given triamcinolone 0.1% cream for his body and desonide 0.05% cream for his face, but the improvement was minimal. After three months of persistent skin lesions, he agreed to try whole-body narrowband UVB therapy twice a week, with significant improvement after few sessions. However, seven months after the onset of his rash he continues therapy as the skin lesions have not resolved completely.

## 2. Discussion

 Imiquimod is a toll-like receptor 7 agonist that stimulates the immune system to produce various proinflammatory cytokines, including interferon-*α* (IFN-*α*), tumor necrosis factor-*α* (TNF-*α*), and various interleukins (e.g., IL-1 and IL-10) [1]. Imiquimod 5% is approved by the FDA for treating actinic keratoses, superficial basal cell carcinomas, and genital warts [2]. While imiquimod is usually well tolerated, it is known to cause localized skin reactions including erythema, crusting, and scaling at the site of application in most patients [3]. It has also been implicated as the cause of widespread cutaneous conditions such as exfoliative dermatitis, psoriasis, erythema multiforme, and hyperpigmentation, presumably through systemic absorption [3]. The newly available imiquimod 3.75% cream (approved March, 2010) is a presumably safer alternative due to its lower concentration. It is currently only indicated for actinic keratoses of the face and scalp [4]. Surprisingly, pharmacological studies suggest that the absorbed levels of topical imiquimod 3.75% may not be lower than that of the 5% formulation, which may potentially result in similar systemic reactions [5]. Because the off-label usage of imiquimod 3.75% is expanding, the side effect profile of this drug needs to be better established. 

We describe a case of acantholytic pityriasis rubra pilaris (PRP) in a previously healthy patient after using imiquimod 3.75% cream for an actinic keratosis. PRP with acantholysis is a rare skin disorder with an unknown etiology or identifiable preceding event [6]. The exact mechanism of the acantholysis is unclear and is thought to be related to a release of proteolytic enzymes in response to altered keratin and plugged acrosyringia in PRP [7].

This association between topical imiquimod and acantholytic dermatosis does not appear to be an isolated case. Imiquimod 5% cream has been previously reported to cause localized or systemic “pemphigus-like” acantholytic dermatitis, with negative DIF studies [8–11]. In other reports, imiquimod 5% use in patients with preexisting PRP has been linked to exacerbation with features of acantholysis [12, 13]. 

The potential for imiquimod to cause acantholysis warrants further investigation as it may pose a risk for development of immunobullous disorders. At the current time, the role of toll-like receptor signaling in acantholytic or bullous processes has not been described. Possible explanation for the role of imiquimod in acantholysis may be a result of the increased levels of proinflammatory cytokines, such as TNF alpha, that ultimately affect intercellular adhesions [14]. Elevated levels of TNF alpha have been demonstrated in patients with both pemphigus and PRP [15]. Use of imiquimod 3.75% in patients should be preceded by assessment of previous skin conditions and risk factors. Patients should be cautioned about the possible adverse side effects such as generalized dermatitis, and be monitored closely for such events.
